# Non-Coding Keratin Variants Associate with Liver Fibrosis Progression in Patients with Hemochromatosis

**DOI:** 10.1371/journal.pone.0032669

**Published:** 2012-03-07

**Authors:** Pavel Strnad, Ozlem Kucukoglu, Mariia Lunova, Nurdan Guldiken, Tim C. Lienau, Felix Stickel, M. Bishr Omary

**Affiliations:** 1 Department of Internal Medicine I, University Medical Center Ulm, Ulm, Germany; 2 Medizinische Klinik mit Schwerpunkt Hepatologie und Gastroenterologie, Charite, Universitätsmedizin Berlin, Berlin, Germany; 3 Department of Medicine I, University of Erlangen-Nuremberg, Erlangen, Germany; 4 Department of Molecular and Integrative Physiology, University of Michigan Medical School, Ann Arbor, Michigan, United States of America; Centro de Investigación en Medicina Aplicada (CIMA), Spain

## Abstract

**Background:**

Keratins 8 and 18 (K8/K18) are intermediate filament proteins that protect the liver from various forms of injury. Exonic K8/K18 variants associate with adverse outcome in acute liver failure and with liver fibrosis progression in patients with chronic hepatitis C infection or primary biliary cirrhosis. Given the association of K8/K18 variants with end-stage liver disease and progression in several chronic liver disorders, we studied the importance of keratin variants in patients with hemochromatosis.

**Methods:**

The entire K8/K18 exonic regions were analyzed in 162 hemochromatosis patients carrying homozygous C282Y HFE (hemochromatosis gene) mutations. 234 liver-healthy subjects were used as controls. Exonic regions were PCR-amplified and analyzed using denaturing high-performance liquid chromatography and DNA sequencing. Previously-generated transgenic mice overexpressing K8 G62C were studied for their susceptibility to iron overload. Susceptibility to iron toxicity of primary hepatocytes that express K8 wild-type and G62C was also assessed.

**Results:**

We identified amino-acid-altering keratin heterozygous variants in 10 of 162 hemochromatosis patients (6.2%) and non-coding heterozygous variants in 6 additional patients (3.7%). Two novel K8 variants (Q169E/R275W) were found. K8 R341H was the most common amino-acid altering variant (4 patients), and exclusively associated with an intronic KRT8 IVS7+10delC deletion. Intronic, but not amino-acid-altering variants associated with the development of liver fibrosis. In mice, or ex vivo, the K8 G62C variant did not affect iron-accumulation in response to iron-rich diet or the extent of iron-induced hepatocellular injury.

**Conclusion:**

In patients with hemochromatosis, intronic but not exonic K8/K18 variants associate with liver fibrosis development.

## Introduction

Intermediate filaments (IFs), together with microtubules and the actin-containing microfilaments constitute the three major components of eukaryotic cytoskeleton [1]. Keratins (*KRT* genes, K proteins) represent the IFs of epithelial cells and are further subdivided into subtypes II (including K1–K8 and K71–K80) and I (K9–K28), which are both required to form obligate heteropolymers [2], [3]. Consequently, each epithelial cell expresses at least one type I and one type II keratin [2], [3]. Adult hepatocytes are unique, in that they contain K8/K18 as their only cytoplasmic IFs, while most other cell types display a more complex keratin composition [3]. The simple hepatocellular keratin expression pattern accounts for the observation that animals either lacking K8/K18 or expressing mutant K8/K18 display a predominant hepatic phenotype [4]. These animal findings spurred human association studies, which identified K8/K18 variants to be overrepresented in patients with cryptogenic and non-cryptogenic liver cirrhosis [4]. Subsequent reports showed that the presence of K8/K18 variants predisposes to liver fibrosis development in patients with chronic hepatitis C and primary biliary fibrosis as well as to adverse outcome of acute liver failure [5], [6], [7]. Keratin variants display ethnicity-specific distribution with K8 G62C/R341H and K8 Y54H/G434S being the most common amino-acid altering variants in Caucasians and African-American patients, respectively [7].

The pathogenesis of keratin variants was supported by the transgenic mice overexpressing K8 G62C variant, which are susceptible to Fas-induced apoptosis and microcystin-induced liver injury [8]. Presence of keratin variants predisposes to selective types of liver insults such as Fas but not TNF-α induced apoptotic injury; and thioacetamide- but not carbon tetrachloride-induced liver fibrosis development [9], [10]. This suggests that the presence of keratin variants may predispose to specific liver diseases, while other liver disorders might be resistant to keratin mutation. To test this hypothesis, we studied the role of keratin variants in patients with hereditary hemochromatosis (HHC).

Hereditary hemochromatosis (HHC) comprises genetic disorders leading to iron overload [11]. The most frequent cause of HHC is the homozygous C282Y mutation in the *HFE* gene, which is as frequent as 1∶200 in Caucasians of northern European descent [11], [12]. Homozygous carriers of this mutation present with elevated transferrin saturation, ferritin and serum iron levels [11], [12]. Epidemiological studies demonstrated that the clinical penetrance of *HFE* mutations is highly variable and does not necessarily lead to a clinically-manifest iron overload or even development of end-stage liver disease [11], [12]. Additional modifying factors including gender, alcohol consumption or co-infection with hepatitis C virus are involved [11], [12]. In addition, polymorphic variants of genes coding for modulators of iron metabolism, oxidative stress and certain cytokines were also shown to affect disease development [13], [14], [15].

In a study that examined candidate gene variants that may associate with the presence of HHC, the K8 R341H heterozygous variant was identified in 3 of 13 iron overload subjects but not in 5 controls [16]. This K8 variant was then examined in 119 subjects with the C282Y/C282Y *HFE* mutation and 116 age-, sex- and ethnic background matched controls. In that group, 9 patients in the HHC cohort carried the K8 R341H variant but none in the controls [16]. However, the frequency of the K8 R341H in more than 800 controls was 3.1% [5]. Notably, iron overload in HHC patients is known to generate reactive oxygen species [17], [18], and earlier findings demonstrate that mice that overexpress the K18 R90C variant manifest upregulation of hepatic oxidative injury gene products under basal conditions [17], [18]. These collective findings led us to analyze a cohort of 162 HHC patients from central Europe for the presence of keratin variants, and to evaluate the impact of iron overload in previously established transgenic mice overexpressing the human K8 G62C variant.

## Methods

### Ethics statement

The study protocol was reviewed and approved by the local ethics committees of the participating centers Austria (Vienna, Salzburg, and Innsbruck) and Germany (Regensburg, Erlangen) between 1991–2005, and all recruited patients gave informed and written consent.

All animal experiments were conducted with the approval of UCUCA under animal protocol number 10081 (Michigan, USA) for animal welfare.

### Hemochromatosis Patient Cohort and Control Subjects

To determine the impact of keratin variants on liver disease development in HHC, we analyzed a previously described cohort of 162 homozygous carriers of the *HFE* C282Y mutation [19], [20]. The analyzed subjects were recruited in five medical centers in Austria (Vienna, Salzburg, and Innsbruck) and Germany (Regensburg, Erlangen) between 1991–2005. The presence of the *HFE* mutation of the analyzed subjects was confirmed by standard genetic testing [21]. All patients underwent liver biopsy and the liver sections were evaluated by two independent pathologists [19], [20] as described by Ishak et al. [22], with fibrosis stages 5 and 6 being considered as cirrhosis. Serum iron, ferritin, and transferrin levels were measured by routine commercial testing. Hepatic iron concentration (HIC) was measured by atomic absorption spectrometry [23], and is expressed as µg/g of dry liver tissue weight. The hepatic iron index (HII) was calculated by dividing the HIC by the age of the patients (in years) and by the molecular weight of iron. Transferrin saturation was calculated as follows: (serum iron×70.9)/(serum transferrin). Reference ranges are: serum iron, 40–150 µg/dL; serum ferritin in men, 18–440 ng/mL; serum ferritin in premenopausal women, 8–120 ng/mL; serum ferritin in postmenopausal women, 30–300 ng/mL; HIC, 300–1400 µg iron/g of dry liver tissue weight; transferrin saturation, 16–45%. The cut-off criteria for significant alcohol consumption were: <20 g/day (females) and <60 g/day (males) [19], [20]. The presence of major liver disorders (e.g., viral hepatitis, heavy alcohol consumption, Wilson disease, primary biliary cirrhosis) was excluded by the appropriate clinical workup [20]. Informed consent was obtained from all subjects and the study was approved by the Ethics Committees of the participating centers. The liver-healthy control subjects were recruited in the medical centers of Bern (Switzerland) and Erlangen (Germany).

### Mutation Detection

Genomic DNA was isolated from peripheral whole blood using an established DNA isolation kit (Qiagen GmbH, Hilden, Germany). For hemochromatosis patients, all 15 K8 and K18 exons and their adjacent exon-intron boundaries were amplified using previously described primers and a mixture of T Taq and Optimase Polymerases (Transgenomic, Omaha, NE) [24], while only K8 exons 1 and 6 were analyzed in control subjects. All amplicons were pre-screened with denaturing high pressure liquid chromatography (DHPLC) [24]. Probes with abnormal elution peak were purified with a QIAquick PCR purification kit (Qiagen) and sequenced in both directions using an ABI PRISM 377 DNA Sequencer (Applied Biosystems, Foster City, CA). The location of the coding *KRT8/KRT18* variants was assigned based on the mRNA sequences NM 002273.2 and NM 000224.2, respectively, while genomic sequences M34482.1 and AF179904.1 were employed for assignment of non-coding variants (www.ncbi.nlm.nih.gov). New genetic variants were reported in GenBank (accession numbers for K8 Q169E, R275W and E339A are rs199422323, rs199422324 and rs144044115, respectively).

### Mouse Experiments

To study whether the presence of keratin variants affects hepatic iron accumulation, previously described transgenic mice (3–4 months old, four male/group) overexpressing human wild-type K8 (K8 WT) or the K8 G62C variant [8] were fed *ad libitum* with 3% carbonyl iron (Sigma-Aldrich, Germany)-containing diet (5001 Labdiet, Brentwood, MO) for one month. Age-matched male mice kept on a standard diet were used as controls. Mice were euthanized by CO_2_ inhalation and blood was collected by intracardiac aspiration to measure serum alanine aminotransferase (ALT) levels. Livers were removed and cut into pieces for: (i) fixation in 10% formaldehyde for histological staining, (ii) snap freezing to measure hepatic iron concentration (HIC), or submerging into RNAlater stabilization reagent (Qiagen) for RNA analysis. Histological images were taken by Olympus BX40 with spot insight QE camera. The mRNA was transcribed into cDNA using oligo-dT primers and Superscript II reverse transcriptase (Invitrogen, Germany). Quantitative real-time polymerase chain reaction (RT-PCR) was carried out with SYBR green Master Mix (Applied Biosystems; Foster City, CA) and performed in duplicate using specific mouse hepcidin (encoded by *Hamp* gene), collagen1 (*Col1a1*), smooth muscle α-actin (*SMA*) and transforming growth factor-β (*TGF-β*) primers and *L7* ribosomal protein as an internal control (Table S2). All animals received humane care and their use was approved by the Institutional Animal Care Committee.

### Determination of Hepatocellular Iron Toxicity

To test whether the presence of keratin variants influences hepatocellular iron toxicity, we isolated primary hepatocytes from K8 WT and G62C males as described before [8]. Briefly, the portal vein was cannulated and perfused with preheated liver Perfusion medium (Gibco 17701, Invitrogen, Darmstadt, Germany) then with pre-warmed 67 µ/mg Worthington type I collagenase-containing DMEM medium (CellSystems Biotechnologie Vertrieb GmbH, Troisdorf, Germany). The cell suspension was collected from extirpated livers, filtered through 100 µm-cell strainers (BD Falcon 9322799, Heidelberg, Germany) and centrifuged at 50 g for 1 min. The pellet was washed three times, and the isolated cells were cultured in hepatocyte culture medium (Gibco Hepatozym-SFM 17705, Darmstadt, Germany) supplemented with 10% FCS, 1% penicillin-streptomycin and L-Glutamine. To mimic the acute iron toxicity, hepatocytes were subjected to 100 µM iron-nitrilotriacetic acid complex (FeNTA) for 48 hours with the molar ratio of NTA/Fe^3+^ of 2∶1 [25], [26]. Untreated cells or cells treated with 100 µM nitrilotriacetic acid (NTA) without Fe were used as controls. Cell viability was determined using a commercially available MTT (3-(4,5-Dimethylthiazol-2-yl)-2,5-diphenyltetrazolium bromide) assay (Roche Cell Proliferation Kit I). Lactate dehydrogenase (LDH) and ALT were measured from cell supernatants.

### Statistical Analysis

The unpaired two-tailed t or chi-square tests were used, and P values less than 0.05 were considered statistically significant.

## Results

### Patient Demographics

The clinical characteristics of the previously described cohort of 162 subjects carrying homozygous *HFE* C282Y mutation are displayed in Table 1; [19], [20]. Most patients were males and harbored a significantly higher risk to develop liver cirrhosis than females (*p<0.0001*). Similarly, all assessed parameters of iron metabolism were markedly higher in males compared to females (Table 1) (ferritin *p<0.0001*, HIC *p = 0.001*, HII *p = 0.003*). Only a minority of patients had significant alcohol consumption.

**Table 1:** Patient Demographics and Biochemical Values.

Demographics	Male	Female	Total	# Patients without data (%)
# of patients (%)	120 (74.5)	41 (25.5)	162 (100)	1(0.6)
Age at LBx, mean ± SD (years)	50±13	52±12	50±13	-
Cirrhosis yes/no (%)	58/60 (49/51)	3/38 (7/93)	61/99(38/62)	-
Alcohol consumption (yes/no)	6/104	4/31	10/135	19 (11.7)
Serum iron (µg/dL), mean± SD	213±43	195±35	208±41	38 (23.5)
Transferrin Saturation (%), mean± SD	87±17	74±19	84±18	29 (17.9)
Serum ferritin (ng/mL), mean± SD**	2501±1953	711±592	2081±1891	17 (10.5)
HIC (µg/g dry weight), mean± SD	13339±8537	7416±6945	11671±8518	59 (36.4)
HII, mean± SD	5.2±3.6	2.9±3.2	4.6±3.7	55 (34.0)

Abbreviations: LBx:liver biopsy; SD: standard deviation; HIC: hepatic iron concentration; HII:hepatic iron index.

### Analysis for Presence of Coding K8/K18 Variants

Genetic analysis of the entire coding regions of K8/K18 identified 10 heterozygous K8 variants that result in amino acid substitution (overall variant frequency 6.2%, Table 2). K8 G62C and K8 R341H were the most frequent amino-acid-altering variants and were found in three and four independent patients, respectively. One patient harbored two heterozygous amino-acid altering K8 variants (K8 A319S+R341H). Two novel variants were detected that had not been described before. One of the novel variants, *KRT8* 505 C→G resulting in a K8 Q169E substitution within the rod domain (subdomain 1B), was found in two patients. The other novel variant, *KRT8* 823 C→T that codes for a K8 R275W substitution in the L2 linker sequence of *KRT8*, was found in one patient. The novel variants were detected in non-cirrhotic patients, and the involved amino acid residues are remarkably conserved among species and among some type II keratins (Figure S1). In addition to amino-acid-altering variants, a previously described common K8 L227L polymorphism was observed (not shown) [27].

**Table 2:** Distribution of Exonic Keratin Variants.

*KRT8*			Cirrhosis (# and % of patient)		Total # of variant (%)	Total # of control (%)
Variant	Nucleotide	Location	Yes (61/38)	No (99/62)	162	234
G62C	GGC→TGC	Exon 1	3	0	3 (1.85)	6 (2.6)
I63V*	ATC→GTC	Exon 1	0	0	0	2 (0.8)
Q169E†	CAG→GAG	Exon 2	0	2	2 (1.23)	N.T.
R275W†	CGG→TGG	Exon 5	0	1	1 (0.62)	N.T.
A319S*	GCT→TCT	Exon 5	0	1$	1 (0.62)	N.T.
E339A†, *	GAG→GCG	Exon 6	0	0	0	1 (0.4)
R341H	CGT→CAT	Exon 6	1	3$	4 (2.50)	9 (3.8)
**# Patients with any variant (%)**			**4 (6.56)**	**6** ^&^ **(6.06)**	**10 (6.17)**	**19 (8.1)**
**# Patients with significant variant (%)**			**4 (6.56)**	**3 (3.03)**	**7 (4.32)**	**15 (6.4)**

The table displays the number of patients with and without liver cirrhosis for the listed keratin variants.

* The highlighted variant is considered to be a “polymorphism” rather than a “mutation” that is likely to have biologic significance.

† Novel variants which were not previously described.

$ One female patient harbored 2 independent amino acid altering KRT8 variants (R341H+A319S). N.T.not tested.

An analysis of K8 variant hotspots in 234 liver healthy Central European subjects revealed a comparable variant frequency of 8.1% with K8 R341H and G62C variants being found in nine and six subjects respectively. One novel variant (E339A) was detected that had not been described before. The frequency of amino-acid-altering variants was similar in cirrhotic and non-cirrhotic patients (6.6% vs. 6.1%). Among the described keratin variants, only K8 G62C and R341H were previously clearly implicated in liver disease development [4] and the frequency of these two variants was somewhat higher in cirrhotic versus non-cirrhotic patients (6.6% vs 3.0%; “significant variants” in Table 2). This was mainly due to the K8 G62C variant, which was found exclusively in patients with liver cirrhosis (Table 2) but the numbers were too small to reach statistical significance.

### Characterization of the Non-Coding K8/K18 Variants

Our genetic analysis identified 9 non-coding heterozygous K8 variants (Table 3). Among these, an intronic KRT8 IVS7+10delC deletion was the most common variant and exclusively associated with K8 R341H substitution as described before [28]. IVS6+46A>T intronic variant was the only intronic variant found in the control group and was seen in one subject (out of 234). The presence of additional intronic variants (IVS7+20G>A and IVS8+31C>T) was not analyzed in the control group. When compound exonic-intronic variants were excluded, “pure” intronic variants (labeled as “total intronic variants in Table 3) were seen in 6 hemochromatosis patients (variant frequency 3.7%). Five patients carrying “pure” intronic variants displayed liver cirrhosis (8.2%), while only one subject did not have cirrhosis. Consequently, the non-coding *KRT8* variants were significantly overrepresented in cirrhotic patients (*p = 0.02*). However, neither intronic nor exonic keratin variants associated with iron accumulation in hemochromatosis patients as suggested by similar levels for serum iron, transferrin, transferrin saturation, ferritin and HIC and HII indices (Table 4). On the other hand, cirrhotic subjects were older and had higher transferrin saturation than non-cirrhotic patients (Table S1).

**Table 3:** Distribution of Non-Coding Keratin Variants.

*KRT8*		Cirrhosis (# and % of patient)		Total # of variant (%)
Variant	Location	Yes (61/38)	No (99/62)	162
IVS7+10delC*	Intronic	1	3	4
IVS6+46A>T†	Intronic	1	1	2 (1.23)
IVS7+20G>A	Intronic	3	0	3 (1.85)
IVS8+31C>T	3′-UTR	1	0	1 (0.62)
**Total intronic variant (%)**		**5 (8.20)** **	**1 (1.01)** **	**6 (3.70)**

The table displays the number of patients with and without liver cirrhosis for the listed keratin variants.

* IVS7+10delC variant completely associates with KRT8 R341H variant and is not included in the count of total intronic variants.

† The highlighted variant was found only in one control subject (out of 234).

** ***p*** **= 0.02**.

**Table 4:** The Clinical Features of Hemochromatosis Patients Harboring Exonic and Intronic Keratin 8 Variants.

	Presence of Exonic Variants		Presence of Intronic Variants	
Demographics	YES (n = 10)	NO (n = 152)	YES (n = 6)	NO (n = 156)
Sex (M/F)	7/3	113/38	5/1	115/40
Age at LBx, mean ± SD (years)	55±11	50±13	59±8	50±13
Cirrhosis (yes/no)	4/6	57/93	5/1	56/98
Alcohol consumption (yes/no)	0/6	10/127	0/6	10/127
Serum iron (µg/dl), mean± SD	216±31	208±42-	202±29	209±42
Transferrin Saturation (%), mean± SD	84±20	84±18	95±6	84±18
Serum ferritin (µg/l), mean± SD	2377±2147	2409±4703	2166±1786	2077±1901
HIC (µg/g), mean± SD	14012±8560	11552±8543	12765±9145	11627±8539
HII, mean± SD	5.4±3.6	4.5±3.7	3.7±2.3	4.6±3.7

Abbreviations: LBx:liver biopsy; SD: standard deviation; HIC: hepatic iron concentration; HII: hepatic iron index.

Note that intronic keratin variants were preferentially found in male subjects (*p = 0.09* for distribution among the sexes).

### The K8 G62C Variant Does Not Alter Experimental Iron Accumulation

**Figure 1:**
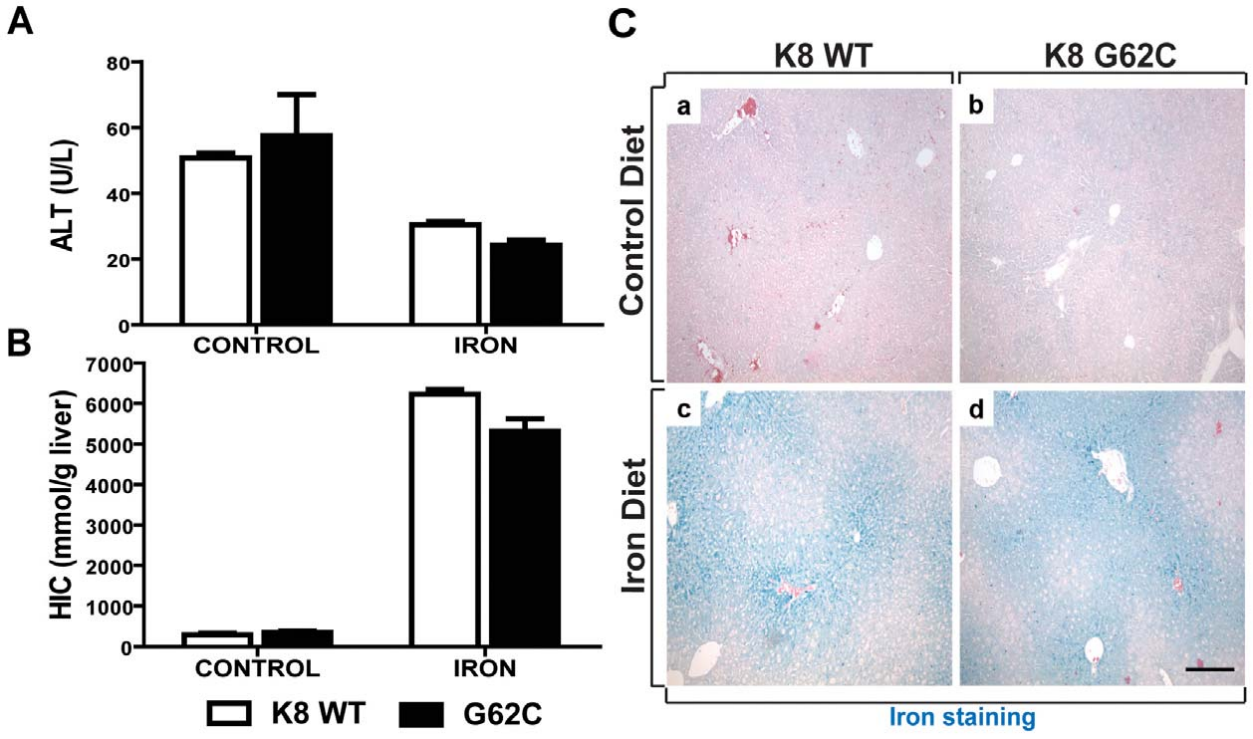
The K8 G62C variant does not influence the extent of hepatocellular iron accumulation. To test, whether hepatocellular iron accumulation is affected by the presence of keratin variants, transgenic mice overexpressing wild-type human keratin 8 (K8 WT) or K8 G62C variant were fed with 3% carbonyl iron-containing diet for one month (Iron) and compared to age-matched animals kept on standard diet (Control) (n = 4 mice/group). Iron-feeding did not lead to an increase in serum ALT (**A**), but caused a significant elevation in hepatic iron concentration (HIC) (**B**). There was no significant difference in ALT or HIC levels between K8 WT and G62C mice after iron feeding. (**C**) Perl's Prussian blue staining revealed a similar pattern of iron deposition in both mouse lines. Scale bar = 200 µm.

Given that all patients harboring the K8 G62C variant displayed liver fibrosis, we used previously-characterized transgenic mice that overexpress K8 G62C and control mice that overexpress WT K8 [8] to test whether K8 G62C affects iron accumulation. As expected, both untreated and iron-fed K8 WT and K8 G62C mice had normal ALT levels (Figure 1A), and did not display any histological signs of liver damage (not shown). Iron-feeding led to a dramatic increase in hepatic iron, which were slightly more pronounced in K8 WT animals; however, the difference between genotypes is not statistically significant (p = 0.08; Figure 1B). Furthermore, both genotypes expressed nearly identical levels of the iron uptake-regulating hormone hepcidin (*Hamp*) and comparable levels of fibrosis-associated genes collagen 1 (*Col1a1*), smooth muscle α-actin (*SMA*) and transforming growth factor-β (*TGF-β*) (Figure S2). In addition, Perl's Prussian blue staining revealed a similar pattern of iron accumulation in both genotypes (Figure 1C).

### The K8 G62C Variant Does Not Increase Mouse Hepatocyte Iron Toxicity Ex-Vivo

**Figure 2:**
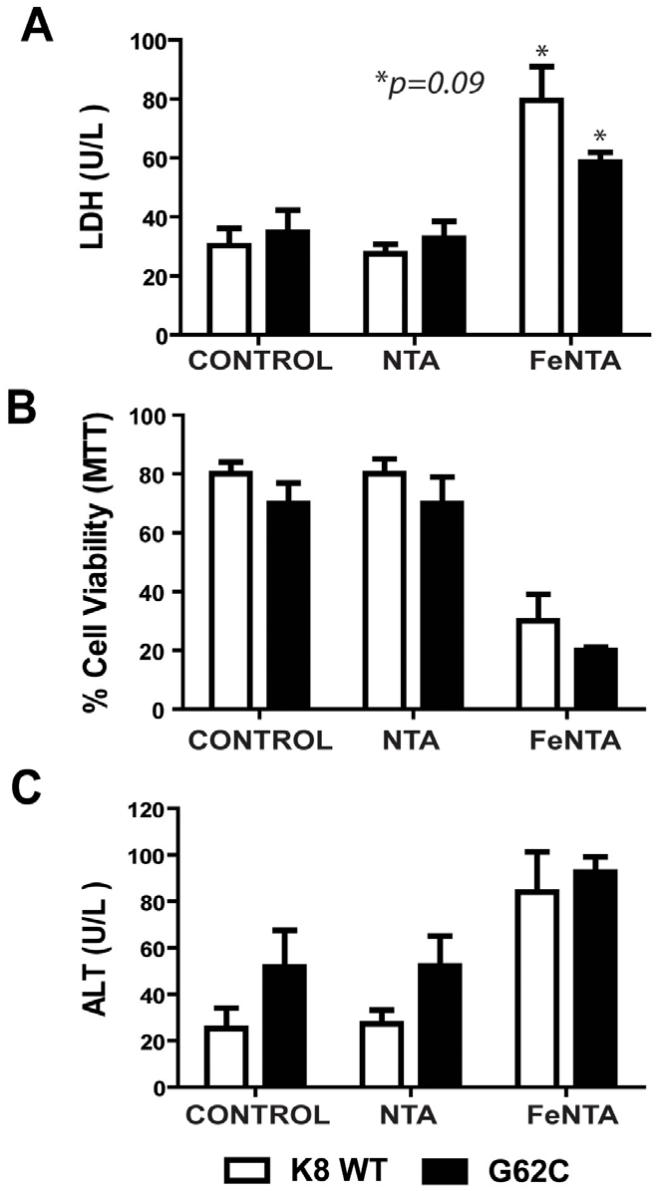
The K8 G62C variant does not affect iron toxicity in ex vivo cultured hepatocytes. To study the hepatocellular iron toxicity, primary hepatocytes from transgenic animals overexpressing K8 WT or K8 G62C were cultured in normal media (Control) or media supplemented with 100 µM nitrilotriacetic acid (NTA) or 100 µM iron-NTA (FeNTA) for 24 hours. FeNTA, but not NTA treatment led to a significant increase in LDH (**A**) and ALT levels (**C**) in cell culture supernatants together with significant decrease in cell viability as assessed by MTT assay (**B**). The enzyme levels and cell viability did not differ significantly between K8 WT and K8 G62C hepatocytes.

To test whether the presence of K8 G62C variant affects hepatocellular iron toxicity, primary hepatocytes from K8 WT and G62C mice were treated with FeNTA. FeNTA exposure led to significant increase in the release of ALT and LDH into the cell culture supernatant; which, however, did not differ significantly between the genotypes (Figure 2A, B). Similarly, addition of FeNTA reduced cell viability in both K8 WT and G62C hepatocytes to a similar extent (Figure 2C), thereby suggesting that the presence of K8 G62C does not enhance hepatocyte iron toxicity.

## Discussion

In the study herein, we first analyzed HHC samples for the presence of K8/K18 variants. Amino-acid-altering keratin variants were detected in 10 of 162 HHC patients (variant frequency 6.2%) and a similar frequency of keratin variants was seen in the control group (8.1%). In contrast, substantially higher keratin variant frequencies (>10%) were previously described in patients with end-stage liver disease or acute liver failure, which is likely due to the enrichment of keratin variants in patients with severe liver disease [5], [7]. On the other hand, comparable keratin variant frequencies were seen in two European studies, which analyzed patients with chronic hepatitis C infection and primary biliary cirrhosis that included patients with all ranges of liver disease severity [6], [28].

Among single point mutations, we identified four patients with K8 R341H variant (variant frequency 2.5%), which is comparable with the frequency seen in liver-healthy Caucasians [7] and hitherto analyzed control subjects (variant frequency 3.8%), but lower than a previously analyzed US HHC patient cohort (variant frequency 7.6%, p = 0.08, [16]). Furthermore, we observed a novel K8 Q169E variant in two unrelated HHC patients, which we had not detected in >1000 subjects analyzed to date with various liver diseases [5], [7]. The K8 Q169E variant introduces a charged amino acid, and leads to replacement of the highly conserved K8 Q169. Given that no mutations have been reported in homologous keratin residues among other type II keratins (http://www.interfil.org/), further studies are needed to address the potential pathogenic role of this variant.

While the amino-acid altering K8/K18 variants exhibited similar frequencies in HHC patients with and without liver cirrhosis, all patients harboring the K8 G62C variant had liver cirrhosis. This mutation might therefore be of particular relevance in HHC. This hypothesis is further supported by the fact that this variant introduces a cysteine into the K8 backbone, which normally lacks cysteines, and leads to keratin crosslinking and impairment of keratin filament reorganization upon oxidative stress [29]. Given the established role of oxidative stress in HHC development as well as the finding that an introduction of cysteine into otherwise cysteine-less K18 predisposes mice to oxidative injury [17], [18], we used previously described transgenic mice overexpressing K8 G62C to study the importance of K8 G62C in iron-overload [24]. However, K8 G62C presence did not accelerate iron accumulation or augment iron-induced hepatocellular damage and apoptosis (Figure 1, 2). We posit that if K8 G62C does indeed predispose to liver disease progression, then it does so in a manner that is independent of iron accumulation.

In summary, the combination of genetic and experimental data suggests that in contrast to hepatitis C, primary biliary cirrhosis and acute liver failure [5], [7], amino-acid altering variants may not contribute to liver disease development in HHC. This finding is not surprising since different liver disorders were shown to posses their unique genetic disease-modifiers [30]. For example, factors modifying iron accumulation might be particularly important in hereditary hemochromatosis, while genes affecting inflammatory response and lipid metabolism may contribute to disease progression in patients with hepatitis C infection and alcoholic/non-alcoholic liver disease, respectively [13]–[15] [30].

Unlike the exonic variants, the intronic variants were shown to be overrepresented in HHC patients with liver cirrhosis. This finding represents the first such liver disease association since intronic K8/K18 variants did not associate with liver fibrosis development in patients with chronic hepatitis C infection [28]. However, intronic variants may very well be functional, given that introns harbour multiple functional elements that regulate alternative splicing or transcription [31]. Furthermore, there was a trend for intronic variants to be more abundant in primary biliary cirrhosis patients as compared with the control group; however their association with liver disease progression was not analyzed [6]. Given the limited number of patients analyzed in the present study and the fact that there are no available data about the biological significance of the observed intronic variants, further studies are needed to determine their role in liver disease.

## Supporting Information

Figure S1 **Conservation of novel K8 variants among species and type II keratins.** The sequences surrounding the amino acids K8 Q169 (A, B) and K8 R275W (C, D) are displayed for selected species (A, C) and type II keratins (B, D). The sequences illustrate the single nucleotide variations leading to the highlighted amino acid change (E, F). Note that K8 Q169 is highly conserved among species and among type II keratins, while K8 R275 amino acid is highly conserved among species, and is also conserved from a charge perspective among type II keratins. The figures were assembled using the following reference sequences: K8: NM 002273.3 (Homo sapiens), NM 19937.1 (Rattus norvegicus), NM 031170.2 (Mus musculus), BC 044116.1 (Xenopus laevis) and NM 200080.2 (Danio rerio); Type II Keratins: NM 006121.3 (*KRT1*), NM 000423.2 (*KRT2a*), NM 015848.4 (*KRT2p*), NM 057088.2 (*KRT3*), NM 002272.3 (*KRT4*), NM 000424.3 (*KRT5*), NM 005554.3 (*KRT6A*), NM 005555.3 (*KRT6B*), NM 005556.3 (*KRT7*).
(TIF)

Figure S2 **mRNA expression of fibrosis-related genes and hepcidin does not differ among K8 WT and G62C fed iron-rich diet.** WT mRNA levels are set at 1.0 and G62C mRNA levels of the indicated transcripts are displayed relative to WT. mRNA was isolated from 4 independent livers per genotype.
(TIF)

Table S1 The clinical characteristics of the previously described cohort of 162 subjects carrying homozygous HFE C282Y mutation (cirrhotic patients vs non-cirrhotic patients).
(DOC)

Table S2 Sequence of primers used for quantitative real time-PCR and length of the amplified products.
(DOC)
