# Supplementary figures and images for: Non-Coding Keratin Variants Associate with Liver Fibrosis Progression in Patients with Hemochromatosis
Source: PLoS One. 2012 Mar 7;7(3):e32669. doi: 10.1371/journal.pone.0032669 (PMC3296740; doi:10.1371/journal.pone.0032669)

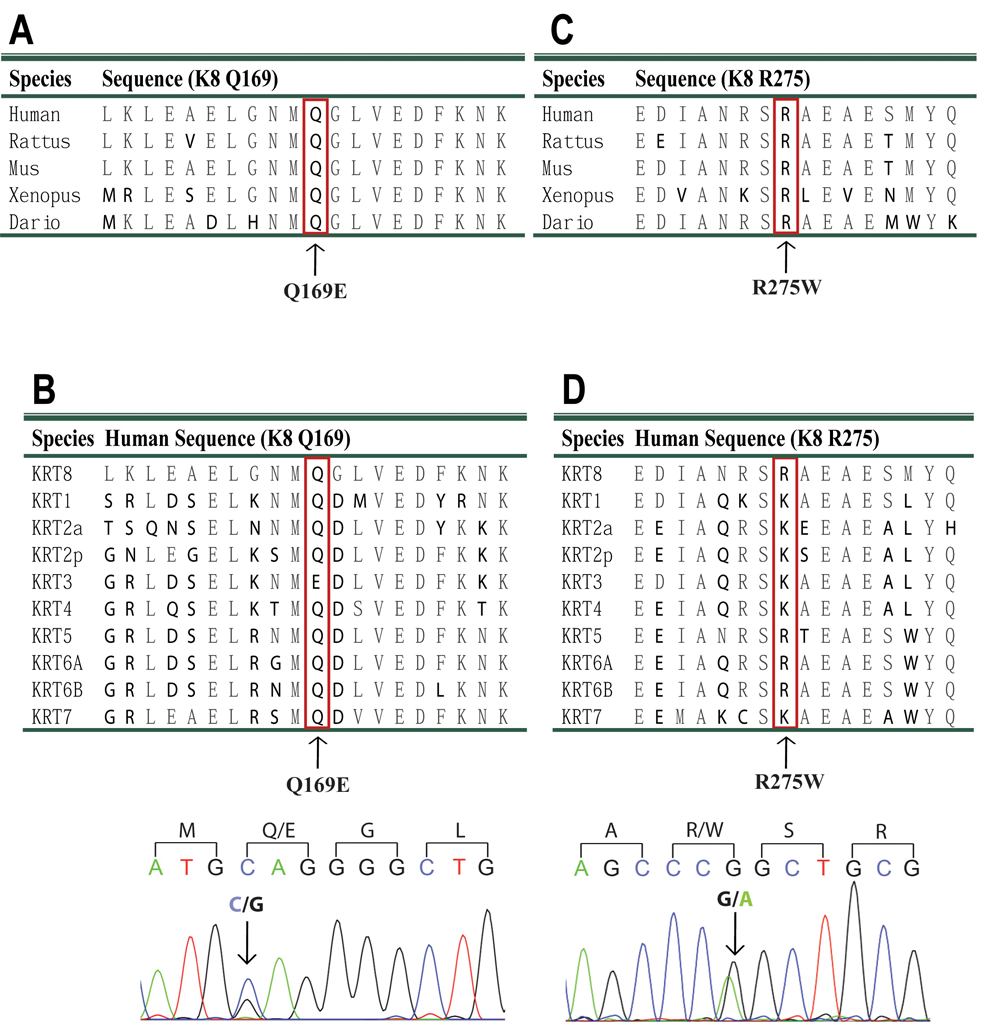

Supplement: Figure S1 — Conservation of novel K8 variants among species and type II keratins. The sequences surrounding the amino acids K8 Q169 (A, B) and K8 R275W (C, D) are displayed for selected species (A, C) and type II keratins (B, D). The sequences illustrate the single nucleotide variations leading to the highlighted amino acid change (E, F). Note that K8 Q169 is highly conserved among species and among type II keratins, while K8 R275 amino acid is highly conserved among species, and is also conserved from a charge perspective among type II keratins. The figures were assembled using the following reference sequences: K8: NM 002273.3 (Homo sapiens), NM 19937.1 (Rattus norvegicus), NM 031170.2 (Mus musculus), BC 044116.1 (Xenopus laevis) and NM 200080.2 (Danio rerio); Type II Keratins: NM 006121.3 (KRT1), NM 000423.2 (KRT2a), NM 015848.4 (KRT2p), NM 057088.2 (KRT3), NM 002272.3 (KRT4), NM 000424.3 (KRT5), NM 005554.3 (KRT6A), NM 005555.3 (KRT6B), NM 005556.3 (KRT7). (TIF) [file pone.0032669.s001.tif]

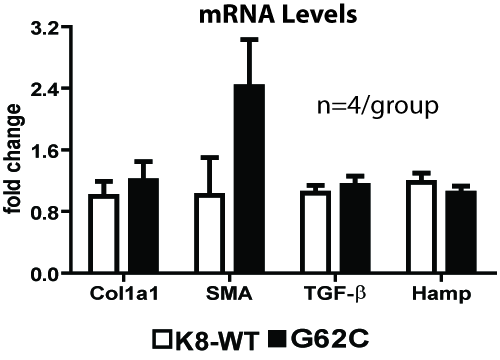

Supplement: Figure S2 — mRNA expression of fibrosis-related genes and hepcidin does not differ among K8 WT and G62C fed iron-rich diet. WT mRNA levels are set at 1.0 and G62C mRNA levels of the indicated transcripts are displayed relative to WT. mRNA was isolated from 4 independent livers per genotype. (TIF) [file pone.0032669.s002.tif]
