# Supplementary material for: Non-Coding Keratin Variants Associate with Liver Fibrosis Progression in Patients with Hemochromatosis
Source: PLoS One. 2012 Mar 7;7(3):e32669. doi: 10.1371/journal.pone.0032669 (PMC3296740; doi:10.1371/journal.pone.0032669)
Supplement: Table S1 — The clinical characteristics of the previously described cohort of 162 subjects carrying homozygous HFE C282Y mutation (cirrhotic patients vs non-cirrhotic patients). (DOC) [file pone.0032669.s003.doc]

| **Table S1.** Patient Demographics and Biochemical Values | | | |
| --- | --- | --- | --- |
| **Demographics** | **Cirrhosis YES** | **Cirrhosis NO** | **# Patients without data (%)** |
| # of patients (Female(%)/Male(%))* | 3 (2) /58 (36) | 38 (24) / 60 (37) | 1 (0.6) |
| Age at LBx, mean ± SD (years) | 57 ± 10 | 46 ± 13 | 1 (0.6) |
| Alcohol consumption (yes/no) | 5/53 | 5/80 | 18 (11.1) |
| Serum iron (μg/dL), mean± SD | 207 ± 34 | 209 ± 45 | 38 (23.4) |
| Transferrin Saturation (%), mean± SD | 91 ± 16 | 79 ± 18 | 29 (17.9) |
| Serum ferritin (ng/mL), mean± SD | 4034 ± 6857 | 1322 ± 1103 | 17 (10.5) |
| HIC (μg/g dry weight) , mean± SD | 13775 ± 8248 | 10540 ± 8505 | 59 (36.4) |
| HII, mean± SD | 4.4 ± 2.6 | 4.6 ± 4.1 | 55 (33.9) |
| Abbreviations: LBx:liver biopsy; SD: standard deviation; HIC: hepatic iron concentration; HII: hepatic iron index.  *Two male patients with hepatocellular carcinoma were not included. | | | |
