# Supplementary material for: Non-Coding Keratin Variants Associate with Liver Fibrosis Progression in Patients with Hemochromatosis
Source: PLoS One. 2012 Mar 7;7(3):e32669. doi: 10.1371/journal.pone.0032669 (PMC3296740; doi:10.1371/journal.pone.0032669)
Supplement: Table S2 — Sequence of primers used for quantitative real time-PCR and length of the amplified products. (DOC) [file pone.0032669.s004.doc]

| **Table S2.** Sequence of primers used for quantitative real time-PCR and length of the amplified products | | | |
| --- | --- | --- | --- |
| **Gene** | **Primers** | **Accession#** | **Size (bp)** |
| *Col1a1* | F: gaagaactggactgtcccaacc  R: gggtccctcgactcctacatctt | NM_007742.3 | 109 |
| *SMA* | F: cctggagaagagctacgaactgc  R: gactccatcccaatgaaagatgg | NM_007392.2 | 108 |
| *TGF-β* | F: gcctgagtggctgtcttttga  R: gctgaatcgaaagccctgtatt‘ | NM_011577.1 | 85 |
| *Hamp* | F: ctgtctcctgcttctcctcct  R: ggctgcagctctgtagtctgt | NM_032541.1 | 84 |
| *L7* | F: gaaaggcaaggaggaagctcatct  R: aatctcagtgcggtacatctgcct | AK017074.1 | 81 |
